# Correction: Unveiling the helicity switching mechanism of a rigid two-tiered stacked architecture

**DOI:** 10.1039/c9ra90007a

**Published:** 2019-02-07

**Authors:** Peng Liu, Yafei Duan, Xihui Bian, Xiaoyao Tan

**Affiliations:** School of Chemistry and Chemical Engineering, State Key Laboratory of Separation Membranes and Membrane Processes, Tianjin Polytechnic University Tianjin 300387 People’s Republic of China liupeng012@tjpu.edu.cn

## Abstract

Correction for ‘Unveiling the helicity switching mechanism of a rigid two-tiered stacked architecture’ by Peng Liu *et al.*, *RSC Adv.*, 2019, **9**, 1501–1508.

The University was omitted in the affiliation in the published article; the revised affiliation is shown here.

The Royal Society of Chemistry apologises for these errors and any consequent inconvenience to authors and readers.

## Supplementary Material

